# Jellyfish for the study of nervous system evolution and function

**DOI:** 10.1016/j.conb.2024.102903

**Published:** 2024-08-20

**Authors:** Karen Cunningham, David J. Anderson, Brandon Weissbourd

**Affiliations:** 1 Department of Biology and The Picower Institute for Learning and Memory, MIT, Cambridge, MA, 02139, USA; 2 Division of Biology and Biological Engineering, Caltech, Pasadena, CA 91125, USA; 3 Howard Hughes Medical Institute, Tianqiao and Chrissy Chen Institute for Neuroscience, Caltech, Pasadena, CA 91125, USA

## Abstract

Jellyfish comprise a diverse clade of free-swimming predators that arose prior to the Cambrian explosion. They play major roles in ocean ecosystems via a suite of complex foraging, reproductive, and defensive behaviors. These behaviors arise from decentralized, regenerative nervous systems composed of body parts that generate the appropriate part-specific behaviors autonomously following excision. Here, we discuss the organization of jellyfish nervous systems and opportunities afforded by the recent development of a genetically tractable jellyfish model for systems and evolutionary neuroscience.

## Introduction

Evolution is the process that created living systems and therefore provides an exceptional window into their functional and organizational logic. An evolutionary perspective encompasses understanding the process of evolution itself [1,2] as well as using comparative approaches as tools to understand biological processes [3]. Rapid advances in neurotechnology make this an exciting time to bring an evolutionary perspective to understanding the neural control of natural behaviors [4,5].

Behavior appeared before the nervous system [6]; this is evidenced by the actions of organisms without neurons, ranging from the navigation of paramecia [7] to the contractions of sponges [8]. These actions rely on a suite of molecular machinery—from ion channels to peptide release—to control cilia, excitable and contractile tissue, and intercellular chemical signaling [6,9,10]. At least once, and perhaps more frequently [11], such machinery became the building blocks for the first neurons and synapses, wiring up to create the first neural networks. Much about the ultimate origins of neurons, synapses, and networks remains unclear [12,13], but from this simple beginning, the incredible diversity of extant animal behaviors and capabilities arose. To what extent this diversity reflects a similar diversity of underlying neural mechanisms versus shared principles remains unclear. Further, when principles are found, it remains uncertain to what extent they are due to homology or convergent evolution. Distinguishing between each of these possibilities has implications for the study of nervous system evolution, function, ecology, bioinspired design, and other fields.

**Figure 1:**
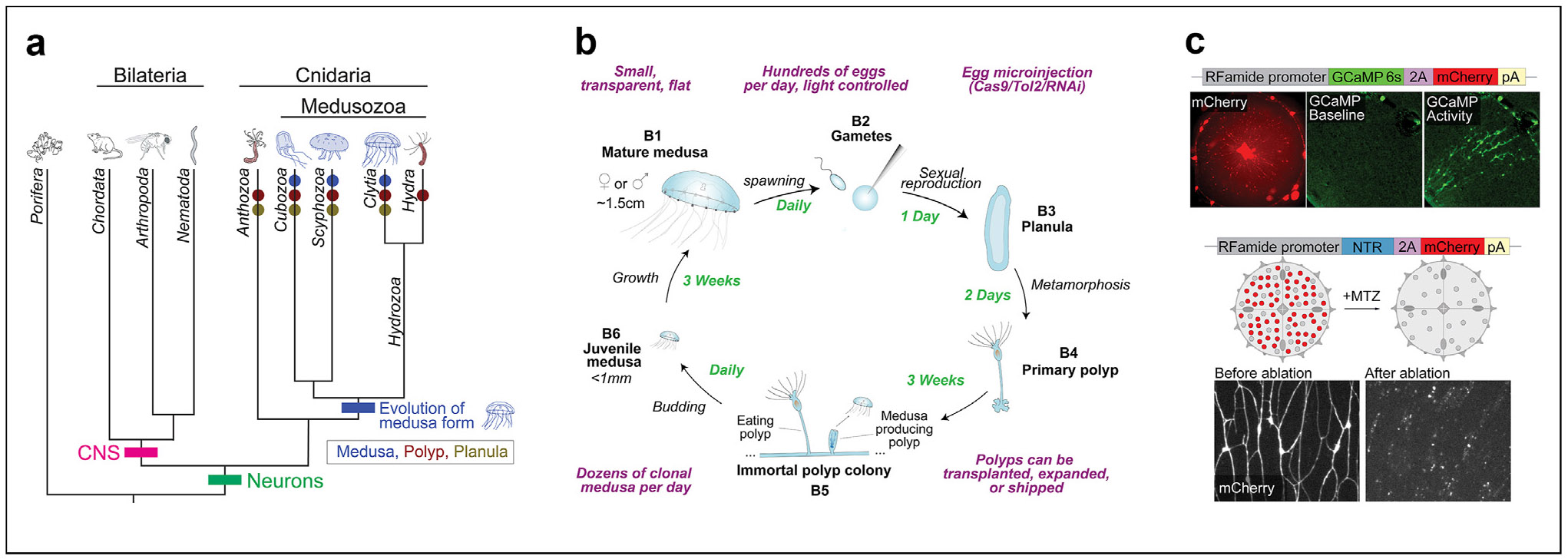
*Clytia hemisphaerica* evolution, lifecycle, and genetic tools. **(a)** Evolutionary relationships among a subset of animals. Neurons and nervous systems emerged prior to the common ancestor of bilaterians and cnidarians and are absent in sponges. Central nervous systems (CNS) evolved in the bilaterian lineage, which includes the primary neuroscience models. The jellyfish (medusa) body form evolved at least once in the medusozoan lineage, with multiple gains and losses of life cycle stages, e.g. in *Hydra*. Branch lengths are not quantitative. **(b)** *Clytia* life cycle. Mature medusae (**B1**) spawn daily 2 h after light onset. Hundreds of eggs (**B2**) are available per day for microinjection with CRISPR/Cas9, Tol2 (for transgenic insertion) or RNAi (for knockdown). A planula larva (**B3**) develops overnight and can be induced to metamorphose the next day. Several days later, a primary polyp (**B4**) forms and expands over several weeks into an immortal polyp colony (**B5**) composed of both feeding polyps (gastrozooids) and medusa-producing polyps (gonozooids) that can bud dozens of clonal medusae per day. Medusae grow from <1 mm (**B6**) to ~1.5 cm in diameter over the next three weeks, until they are sexually mature and begin spawning. *Clytia* polyps can be conveniently maintained, transplanted, expanded, and shipped to other labs. **(c)** *Clytia* transgenic and imaging tools. Top: *Clytia* expressing GCaMP and mCherry under the control of the RFamide promoter; mCherry, and GCaMP activity can be seen in the subumbrellar RFamide network as well as in the nerve rings and mouth. Bottom: *Clytia* expressing the Nitroreductase (NTR) enzyme and mCherry in RFamide neurons. NTR promotes apoptosis in the presence of the drug MTZ, resulting in the inducible, genetic ablation of NTR-expressing neurons.

Cnidaria, the phylum of marine invertebrates that includes corals, anemones, jellyfish, and their relatives (Figure 1a), present exceptional comparative neuroscience opportunities [14]. From a systems neuroscience perspective, many organisms within this phylum are tiny and transparent, with relatively simple behaviors and nervous systems, allowing for whole-animal, all-optical interrogation [15-18]. From an evolutionary perspective, they are an outgroup to the major models in systems neuroscience, all of which are bilaterians (Figure 1a), from which they branched after the evolution of neurons [19]. This position grants cnidarians homologous neurons yet dramatically different, decentralized nervous system organizations, affording a unique perspective on the diversity, origins, and early evolution of neural systems. There is also remarkable diversity within cnidaria, presenting opportunities to use them as a “model clade” [4] to study the evolution of novel morphology and behavior. Lastly, many have unique and poorly understood capabilities, perhaps best exemplified by the “immortal jellyfish,” *Turritopsis* [20], or the freshwater polyp, *Hydra*, which is able to regenerate entirely following dissociation to single cells and reaggregation [21]. This is an exciting time for cnidarian neuroscience, with rapid development of multiple cnidarian neuroscience models, including the exciting, emerging field of systems neuroscience using the *Hydra* polyp [14,16-18,22,23]. Work using *Hydra* is beyond the scope of this brief review, where we focus instead on cnidarian species that have a free-swimming medusa stage.

Cnidarians have complex life cycles that include multiple, distinct stages [24]. Corals and anemones often have larval and polyp stages; in the medusozoans, a medusa (jellyfish) stage subsequently evolved [24], likely to facilitate gamete dispersal (Figure 1a-b). This invention created one of the first freely swimming animal predators on earth [25]. Jellyfish have evolved the most complex behaviors within the cnidarians: they navigate in 3-dimensions in the water column, capture and consume prey, and escape from threats [26-28]. Despite lacking a central brain, previous work has described courtship behaviors [29], sleep states [30], and other complex actions. Jellyfish have also attracted attention due to their remarkable regenerative abilities [31,32], tremendous ecological and socioeconomic impacts [33-35], and status as the most efficient swimmers, inspiring new designs in the rapidly evolving field of biomimetic robotics [36]. In addition to their potential as models for addressing fundamental questions in systems and evolutionary neuroscience, such factors highlight the importance of better understanding jellyfish neurobiology. However, previous work has been severely limited by the lack of genetic tools in any jellyfish species. Here, we discuss the development of a cnidarian species with a jellyfish stage as a genetically tractable laboratory model [15], some of the opportunities that this model affords, and expectations about how its radically different nervous system may be organized to generate behavior.

## *Clytia*: a model jellyfish

The jellyfish *Clytia hemisphaerica* was recently adapted into a genetic neuroscience model in the laboratory of David Anderson at Caltech [15]. *Clytia* has been a fruitful model for several decades for the study of evolution, embryology, regeneration, and other fields [37], and presents an exciting, tractable neuroscience platform: they are small (<1 mm to ~1.5 cm), planar, and transparent, with complex behaviors but compact nervous systems (~ 10,000 neurons in a 1 cm animal [38]). This places them at intermediate numerical complexity compared to major, existing, transparent bilaterian models (*C. elegans* and zebrafish) [39]. Genetic and genomic tools are rapidly being developed: the genome has been sequenced and assembled [24] and whole-animal single-cell RNA-seq (scRNAseq) datasets are available [38]. Multiple forms of knockdown and knockout are routine [40,41] as well as stable transgenesis to deliver reporters and effectors to specific cell types [15] (Figure 1c). This makes *Clytia* the only jellyfish species with such tools available. Together, these features allow for simultaneous, whole-organism optical experimentation in intact, behaving animals using genetic techniques.

The *Clytia* life cycle was a determining factor when it was originally chosen as a laboratory model (Figure 1b) [37,42]. Briefly, jellyfish have separate sexes with synchronous, prolific, daily spawning triggered by the onset of light, affording easy access to embryos for manipulation and genetic crosses of defined parentage (Figure 1B1-B2). An embryo develops into a larva (Figure 1B3) that can be induced to metamorphose into a “primary polyp” (Figure 1B4) by adding a neuropeptide to the water. Each primary polyp generates a vegetatively self-propagating colony of clonal polyps that grows on microscope slides and can be passaged and maintained indefinitely (Figure 1B5). Dozens of clonal jellyfish are released daily from mature polyp colonies (Figure 1B6). Polyps can be transplanted to expand colonies or shipped to share lines. Total generation time is ~7 weeks. While we focus here on the medusa stage, *Clytia’s* inclusion of all three life stages (larva, polyp, and medusa) presents exciting opportunities to study the evolution and development of novel body forms and their corresponding neural systems and behavioral repertoires. For example, the neural control of the larval search and settlement process represents a poorly understood search algorithm with significant ecological implications, i.e. by influencing organismal spatial densities [43]. These life cycle features, combined with the powerful emerging genetic and imaging toolkit, highlight the potential for *Clytia* to become a widely used, genetically tractable model complementing existing cnidarian models.

## The basic organization of jellyfish nervous systems

Jellyfish are incredibly diverse and exhibit deep phylogenetic branching. For example, the last common ancestor of hydrozoan jellyfish, such as *Clytia*, and scyphozoan jellyfish, such as *Aurelia* (moon jellyfish), may have lived more than 500 million years ago [44]. Scyphozoan and hydrozoan jellyfish have dramatically different nervous system organizations, most strikingly demonstrated in the “fundamental experiment” of 1882 [45]. This experiment showed that while tiny pieces of hydrozoan jellyfish will continue to swim following excision, most pieces of scyphozoan jellyfish do not. This is because the swim generating units of scyphozoans have been condensed into several specific regions, called rhopalia, that are required for swimming [46]. More broadly, hydrozoan body parts are able to perform region-specific behavioral subroutines autonomously after they are excised, yet they participate in more complex behaviors when they are coupled together in the intact organism [15]. This observation highlights the robust, decentralized organization and distributed processing of hydrozoan systems and presents opportunities to examine how regions of the nervous system communicate and coordinate to control organismal functions.

**Figure 2:**
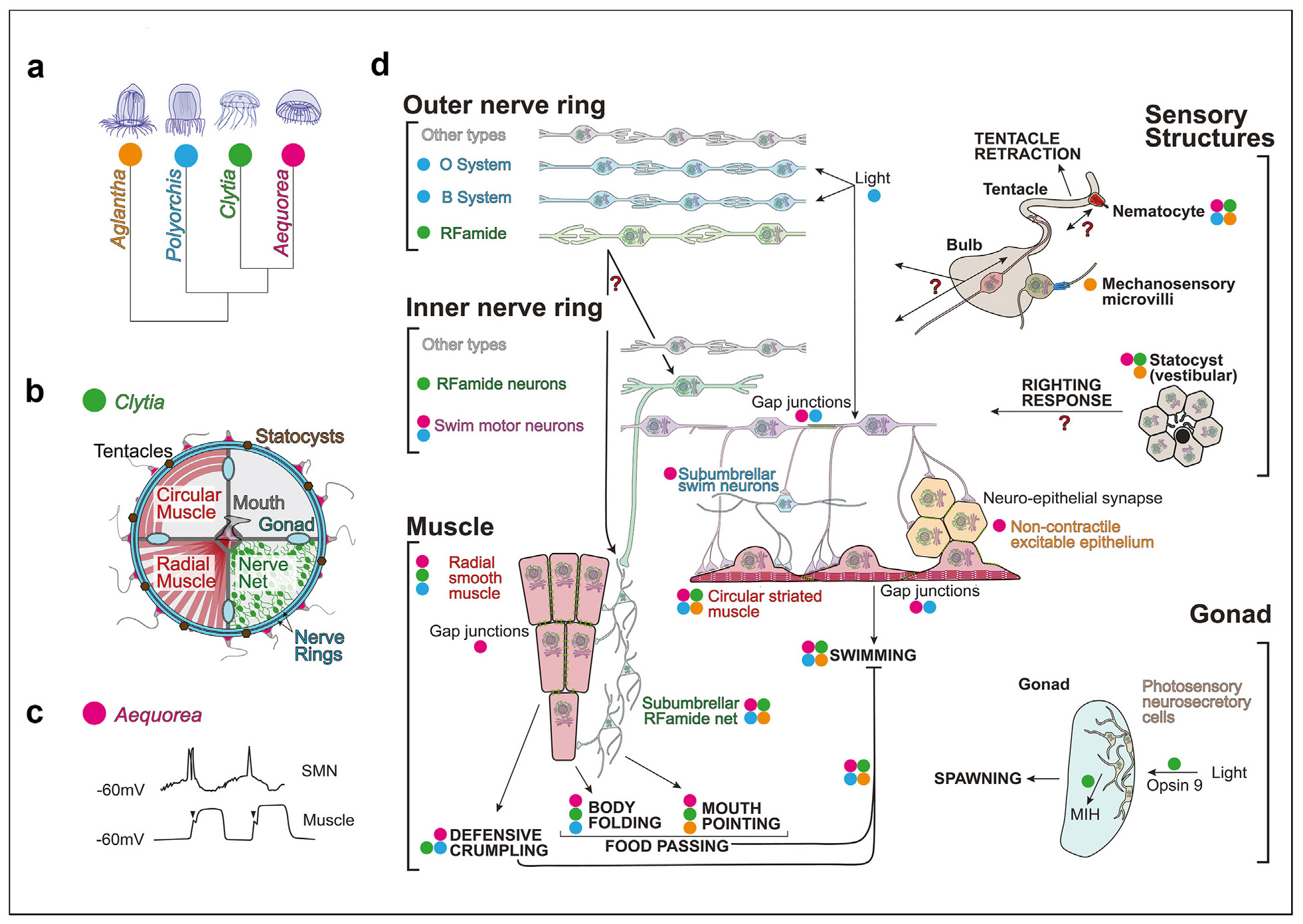
Organization of hydrozoan nervous systems. **(a)** This model summarizes findings from four model hydrozoan jellyfish: *Aglantha* (orange), *Polyorchis* (blue), *Clytia* (green), and *Aequorea* (pink). Colored dots next to cell types and their functions (in d) indicate data from one or multiple of these organisms. The evolutionary relationship between these models is shown [86]. Branch lengths are not quantitative. **(b)** Basic *Clytia* anatomy: a majority of the *Clytia* nervous system is highly condensed into inner and outer nerve rings, which circle the perimeter. Multiple diffuse nerve nets cover the inner umbrella, innervating circular striated muscle and radially oriented smooth muscle. The mouth is in the center of the umbrella, and gonads are located along each gastrovascular canal. The margin is studded with tentacles and gravity-sensing statocysts (adapted [15]) **(c)** Cartoon depiction of electrophysiological properties of swim motor neurons and circular myoepithelium, based on data from *Aequorea* [55,61-63]. An electrically coupled network of “swim motor neurons” in the inner nerve ring functions as pacemakers; their baseline membrane potential oscillates, initiating spiking at the apex of oscillations, modulated by small synaptic input from other neuron populations. These spikes initiate longer square pulse action potentials in the circular striated myoepithelium. Small synaptic potentials precede each muscle spike (denoted by triangles), perhaps representing input from a subumbrellar swim network innervating the muscle sheet **(d)** Model of a hydrozoan nervous system. Colored dots indicate findings from one of the four hydrozoan jellyfish, but a missing dot does not indicate a particular function or feature was found to be lacking from a specific organism. The swim system in *Aglantha* is excluded from this model due to Aglantha’s unusual escape swimming circuitry [46]. The outer nerve ring (top left) includes sensory systems such as the light-sensitive *“O”* and “B” systems in *Polyorchis* that mediate the “shadow reflex” [65,67], an RFamide subnetwork (green), and likely other subnetworks (gray). The inner nerve ring (middle) also includes an RFamide subnetwork (green), swim motor neurons (purple) [48,55,61,62,72], and likely other subnetworks (gray). The inner umbrella (bottom left) includes radial smooth muscle [87] (left) that can either form a sheet (as in *Clytia* [15]) or condense into bands along the canals, as in *Polyorchis* [88] and *Aequorea* [80]. Radial musculature enacts defensive crumpling via global activation [56] as well as local body folding during food passing, which is mediated by the subumbrellar RFamide neurons [15]. *Aglantha* lacks both crumpling and margin-folding and its radial muscle is restricted to the mouth [50]. *Circular*, striated muscle (right) mediates swimming. The two muscle sheets are separate from one another, but are internally gap junction coupled [55]. Noncontractile, excitable epithelial cells can also be electrically coupled to muscle cells (yellow) [61,63]. The swim motor neurons are modulated by multiple types of sensory input and are inhibited during feeding [15,50,54,61,80] and defensive crumpling [56]. Sensory structures (top right) include tentacles that receive chemosensory and mechanosensory information (via hair-cell-like microvilli) [58] and statocysts [57,59], which function as a vestibular system, initiating a “righting” response. Nematocytes (stinging cells) cover tentacles and other areas of the body [89]. The gonad (bottom right) is directly regulated by light, via Opsin-9, which stimulates release of oocyte maturation-inducing hormone (MIH) from photosensory neurosecretory cells, initiating spawning [68-70].

While a neural systems-level understanding of *Clytia* is still in its early stages, we can make predictions based on over a century of anatomical, electrophysiological, and behavioral experiments in related hydrozoan jellyfish species (Figure 2a) [26]. Broadly, hydrozoan jellyfish are expected to loosely follow a common body plan and nervous system organization. They are radially symmetric, with the mouth at the center of the body (Figure 2b) and most of the nervous system concentrated into parallel bundles of neurites that form rings that run around the margin (“nerve rings”). These are generally observed as two bundles, termed an inner and an outer nerve ring, each of which can have multiple subsystems (see below). The remaining neurons are distributed across the animal, forming “nerve nets” that cover the subumbrella, mouth, and tentacles (Figure 2b). However, it remains unclear how well such features will generalize across species: indeed, the diversity of behavior and nervous system organization in this clade of small, transparent organisms presents many opportunities for high-resolution studies of nervous system and behavioral evolution (Box 1) [47,48].

Previous work often describes hydrozoan “nerve nets” as diffuse and unstructured, as they appear so anatomically. However, the first study to use modern genetic tools in a jellyfish, applied to a particular *Clytia* subnetwork, has already revealed unexpected organizational complexity [15]. In that study, genetic ablation of a subpopulation of neurons (defined by expression of an RFamide neuropeptide) revealed that they were specifically required for a feeding action in which directional, inward folding of the margin is combined with mouth pointing to pass food from a tentacle to the mouth. Calcium imaging revealed that ensembles of RFamide neurons generated radially oriented columns of synchronous activity at the site of inward folding, with neural network modeling predicting that this activity could only be explained by a mixture of an underlying intrinsic columnar structure and distance-dependent connectivity. This first systems-level glimpse of jellyfish neural networks in action using modern genetic tools indicated an unap-preciated degree of organizational complexity, much of which likely remains to be discovered. Indeed, RFamide neurons comprise only 10% of all *Clytia* neurons, with scRNAseq and anatomical studies identifying more than a dozen putative neural subtypes organized into distinct, intermingled subnetworks of unknown organization and function [38].

## The nerve rings as the jellyfish central nervous system and the neural control of swimming

Broadly, the nerve rings are thought to be the central coordinators of jellyfish actions [49]. Consistent with this, calcium imaging and manipulations of the *Clytia* RFamide subnetwork demonstrated that RFamide neurons in the nerve rings act upstream of the nerve net during food passing [15]. The nerve rings are expected to be composed of many distinct subsystems: in *Aglantha*, 12 neural subsystems have been described in the nerve rings based on electrophysiological and anatomical studies [50,51]. In *Clytia*, scRNAseq and *in situ* hybridization have shown that the nerve rings may contain the highest diversity of transcriptomically distinct cell types versus other regions [38]. However how these molecular types map to behavioral function is almost entirely unknown in any jellyfish species. In *Clytia*, for example, the molecular identities of neurons controlling swimming behaviors have yet to be identified, as RFamide neurons were neither active during nor necessary for swimming [15].

Swimming behaviors form the core of the jellyfish behavioral repertoire [28] and are controlled principally by the nerve rings. When swimming, jellyfish navigate via body contractions of varying shape, with shape determining fluid interactions and translating into straight versus turning movements [36]. How these shapes are generated remains unclear, but may rely on the degree of synchrony of coupled, pulse-generating units distributed around the nerve rings (see below). Jellyfish, however, are not always swimming; swimming behaviors appear in bouts of starting and stopping, a pattern that is thought to be a form of fishing [52,53]. Swimming behaviors stop during food passing [15,54,55] and protective defensive behaviors [56], and are modulated by diverse sensory modalities, including vestibular statocysts [57-59]. These and other observations raise a series of fundamental neuroscience questions, concerning multisensory integration, motor control, and mechanisms underlying switches between distinct swimming, defensive, reproductive, and feeding behavioral states. For example, despite the clear organismal-level coordination of swimming, swimming implementation appears modular: small wedges of hydrozoan jellyfish are sufficient to generate bouts of swim contractions. This indicates that pulse-generating units of unknown form are densely distributed around the nerve ring, with organismal behaviors arising from their coupling rules; this raises the question of how coordinated organismal behaviors and internal states emerge from such distributed, local interactions.

Much of what we can expect about the neural control of swimming in jellyfish comes from the genus *Aequorea* (Figure 2c), famous as the source of green fluorescent protein [60]. In *Aequorea*, swim pulses are thought to arise from “swimming motor neurons” (SMNs) in the inner nerve ring, which are electrically coupled [48,55,61-63]. SMNs appear to act as pacemakers for swimming as their resting membrane potential oscillates, resulting in short bursts of spiking at the rise of oscillations. SMNs also receive small, graded potentials from synaptic inputs [61]. SMNs then excite a gap junction-coupled sheet of excitable but noncontractile epithelial cells, which are gap—junction coupled to contractile, excitable, striated myoepithelial muscle cells. These myoepithelial cells form an electrically coupled sheet that encircles the animal. The spread of activity around the bell has been suggested to be supplemented by a subumbrellar network of swim neurons, with coincidence detection and filtering potentially also being performed at the level of the epithelial cells, perhaps serving to gate the initiation of swim pulses and control their shape [46,48,55] (Figure 2c, d).

Swimming has been shown to be modulated by diverse chemical and mechanical stimuli, vestibular systems, and light (Figure 2d, right). For example, ciliated hair cells on the surface of tentacles in *Aglantha* send mechanosensory information to the outer nerve ring, impacting the escape response and feeding behavior [58]. The gravity-sensing statocyst can detect motion and orientation to initiate a “righting response” [55,57,59]. Finally, jellyfish can respond to light responses by phototaxis *(Sarsia)* [64], shadow-induced swimming (*Polyorchis*) [65-67], and light-induced spawning (*Clytia*) [68-70]. Photoreception can occur via extraocular photosensory cells or dedicated light-sensing ocelli [71]. To what extent these properties will be found to generalize across species of jellyfish is not yet clear. Further, how information from single and multiple sensory modalities is represented in population neural activity and how this information interacts with the current states of the SMNs and other subnetworks to generate these diverse motor outputs remain unknown at the systems level in any species.

Hydrozoan jellyfish have multiple classes of behaviors that act antagonistically with swimming, including feeding and defensive behaviors. In defensive “crumpling” behavior, mechanical stimuli lead to cessation of swimming and global activation of the radial muscle, causing the periphery of the bell to fold inwards toward the mouth [56]. SMNs have been shown to receive hyperpolarizing (inhibitory) input during defensive crumpling [56,72]. Food passing relies on local rather than global activation of the radial muscle and also corresponds to cessation of swimming in multiple species, including *Clytia* [15,55], with inhibition of SMNs reported in *Polyorchis* [54]. The neural systems-level mechanisms as well as which neurotransmitters are used to generate these interactions, however, remains unclear: broadly, the question of neurotransmitter usage amongst the cnidarians is an important and unresolved question in nervous system evolution.

## Conclusions

Until recently, modern genetic tools could only be used in a small number of organisms. New technologies have changed this, leading to a revolution in the ability to match new model organisms to specific questions and to more broadly explore the diversity of life. However, significant, shared challenges remain, for example, in the ability to rapidly and accurately target effectors to specific cell types, ideally via targeted genomic integration. This is a moment when a concerted, community effort could greatly accelerate the field, focused on developing broadly applicable genetic tools for new models and establishing frameworks for comparing network form and function at the systems level [73].

Here, we have discussed some of what is known of the neural control of behavior in jellyfish and questions that can be addressed by studying these remarkable animals. Our studies demonstrate that neural population imaging at the whole-organism scale can reveal emergent properties of functional network organization that would not be apparent from traditional single-unit recordings or anatomical studies. We have also described *Clytia* as a new, genetically tractable platform to study nervous system evolution and function in a clade of organisms with growing economic and ecological importance. As we continue to develop new tools and resources, we believe that *Clytia* will prove a powerful platform for both universal neuroscience questions and those that arise from *Clytia’s* remarkable capabilities, such as for neural regeneration, and we are eager to support labs that may be interested in adopting *Clytia* for their research program.

## Data Availability

No new data were used for the research described in this review article.

## Box 1. Evolutionary diversity within hydrozoan jellyfish

Though hydrozoan nervous systems share many core features (Figure 2), evolutionary novelties can embellish this basic system to allow for unique capabilities. An example is the escape swim system of the jellyfish medusa *Aglantha digitale*, which is mediated by a single, multinucleated “ring giant axon” in the outer nerve ring, which synapses on “motor giants” in the subumbrella as well as “tentacle giants” in the tentacles that, together, initiate an escape response involving both swimming and tentacle behaviors [51,74-76]. These giant axons are (to our knowledge, uniquely to cnidarians) able to conduct two separate action potentials – a strongly depolarizing sodium spike, which triggers escape swimming, and a slower calcium spike, which mediates slow swimming. Each spike inhibits the other, allowing their guaranteed independence [77,78].

Feeding behaviors also show a range of interesting diversity, as discussed elsewhere [64,79-83]. In *Clytia*, feeding involves both directional umbrella folding and mouth pointing to transfer food from a tentacle located at the margin to the central mouth. In contrast, *Aglantha* uses only pointing of an elongated mouth (“mouth pointing”) without margin folding to retrieve prey from the tentacles [50]. In both *Clytia* and *Aglantha*, RFamide neurons appear to control this behavior. However, in *Clytia*, the RFamide neurons form a diffuse nerve net, whereas in *Aglantha* they have been condensed into several discrete bundles of axons [15,50]. The presence of RFamide in the relevant nerve bundles may suggest a conserved role for the peptide in feeding behavior, independent of the details of the physical implementation.

Feeding behavior that involves mouth pointing in combination with margin folding has also been described in related jellyfish species, such as *Aequorea* [61] and multiple species of narcomedusae [84]. However, other species of hydrozoans have been described as using tentacle bending to pass food directly to the mouth, or simultaneously contracting all of the radial muscle, more similar to crumpling behavior, rather than by directional folding [81,85]. These data demonstrate a rich diversity in how food passing behavior is implemented across jellyfish species. The evolutionary substrate for this diversity, and whether it involves modifications to the RFamide system, remains to be determined. With this remarkable diversity of behavior and nervous system organization in a clade of tiny, transparent organisms, hydromedusae present exciting opportunities for evolutionary systems neuroscience looking forward.

## References

[1] Seeholzer LF, Seppo M, Stern DL, Ruta V: Evolution of a central neural circuit underlies Drosophila mate preferences. Nature 2018, 559:564–569. 29995860 10.1038/s41586-018-0322-9 PMC6276375

[2] McLean CY, : Human-specific loss of regulatory DNA and the evolution of human-specific traits. Nature 2011, 471:216–219. 21390129 10.1038/nature09774 PMC3071156

[3] Yartsev MM, Ulanovsky N: Representation of three-dimensional space in the Hippocampus of flying bats. Science 2013, 340:367–372. 23599496 10.1126/science.1235338

[4] Jourjine N, Hoekstra HE: Expanding evolutionary neuroscience: insights from comparing variation in behavior. Neuron 2021, 109:1084–1099. 33609484 10.1016/j.neuron.2021.02.002

[5] Yartsev MM: The emperor’s new wardrobe: rebalancing diversity of animal models in neuroscience research. Science 2017, 358:466–469. 29074765 10.1126/science.aan8865

[6] Jékely G: Origin and early evolution of neural circuits for the control of ciliary locomotion. Proc. R. Soc. B Biol. Sci 2010, 278:914–922. 10.1098/rspb.2010.2027 PMC3049052 21123265

[7] Brette R: Integrative neuroscience of paramecium, a “swimming neuron.”. eNeuro 2021, 8. ENEURO.0018-21.2021. 10.1523/ENEURO.0018-21.2021 PMC8208649 33952615

[8] Parker GH: The reactions of sponges: with a consideration of the origin of the nervous system. Museum of Comparative Zoölogy at Harvard College 1910, 204.

[9] Arendt D: The evolutionary assembly of neuronal machinery. Curr Biol 2020, 30:R603–R616. 32428501 10.1016/j.cub.2020.04.008

[R10] Musser JM, : Profiling cellular diversity in sponges informs animal cell type and nervous system evolution. Science 2021, 374:717–723. 34735222 10.1126/science.abj2949 PMC9233960

[11] Moroz LL, : The ctenophore genome and the evolutionary origins of neural systems. Nature 2014, 510:109–114. 24847885 10.1038/nature13400 PMC4337882

[12] Colgren J, Burkhardt P: The premetazoan ancestry of the synaptic toolkit and appearance of first neurons. Essays Biochem 2022, 66:781 –795. 36205407 10.1042/EBC20220042 PMC9750855

[R13] Burkhardt P, : Syncytial nerve net in a ctenophore adds insights on the evolution of nervous systems. Science 2023, 380:293–297. 37079688 10.1126/science.ade5645 PMC7617566

[14] Bosch TCG, : Back to the basics: Cnidarians start to fire. Trends Neurosci 2017, 40:92–105. 28041633 10.1016/j.tins.2016.11.005 PMC5285349

[R15] Weissbourd B, : A genetically tractable jellyfish model for systems and evolutionary neuroscience. Cell 2021, 184:5854–5868.e20. 34822783 10.1016/j.cell.2021.10.021 PMC8629132

[R16] Dupre C, Yuste R: Non-overlapping neural networks in Hydra vulgaris. Curr Biol 2017, 27:1085–1097. 28366745 10.1016/j.cub.2017.02.049 PMC5423359

[17] Badhiwala KN, Primack AS, Juliano CE, Robinson JT: Multiple neuronal networks coordinate Hydra mechanosensory behavior. Elife 2021, 10, e64108. 34328079 10.7554/eLife.64108 PMC8324302

[R18] Yamamoto W, Yuste R: Peptide-driven control of somersaulting in Hydra vulgaris. Curr Biol 2023, 33:1893–1905.e4. 37040768 10.1016/j.cub.2023.03.047

[19] Arendt D, Tosches MA, Marlow H: From nerve net to nerve ring, nerve cord and brain — evolution of the nervous system. Nat Rev Neurosci 2016, 17:61–72. 26675821 10.1038/nrn.2015.15

[20] Bavestrello G, Sommer C, Sará M: Bi-directional conversion in Turritopsis nutricula (Hydrozoa). Sci Mar 1992.

[21] Lovas JR, Yuste R: Ensemble synchronization in the reassembly of Hydra’s nervous system. Curr. Biol. CB 2021, 31:3784–3796.e3. 34297913 10.1016/j.cub.2021.06.047

[R22] Botton-Amiot G, Martinez P, Sprecher SG: Associative learning in the cnidarian Nematostella vectensis. Proc Natl Acad Sci USA 2023, 120, e2220685120. 36940325 10.1073/pnas.2220685120 PMC10068830

[R23] Wang H, : A complete biomechanical model of Hydra contractile behaviors, from neural drive to muscle to movement. Proc Natl Acad Sci USA 2023, 120, e2210439120. 36897982 10.1073/pnas.2210439120 PMC10089167

[24] Leclère L, : The genome of the jellyfish Clytia hemisphaerica and the evolution of the cnidarian life-cycle. Nat. Ecol. Evol 2019, 3:801 –810. 30858591 10.1038/s41559-019-0833-2

[25] Cartwright P, : Exceptionally preserved jellyfishes from the middle cambrian. PLoS One 2007, 2, e1121. 17971881 10.1371/journal.pone.0001121 PMC2040521

[26] Meech RW: Electrophysiology and behavior of Cnidarian nervous systems. In Oxford research encyclopedia of neuroscience. Oxford University Press; 2019, 10.1093/acrefore/9780190264086.013.146.

[27] Mackie GO: Central neural circuitry in the jellyfish Aglantha. Neurosignals 2004, 13:5–19. 15004422 10.1159/000076155

[28] Costello JH, Colin SP, Dabiri JO: Medusan morphospace: phylogenetic constraints, biomechanical solutions, and ecological consequences. Invertebr Biol 2008, 127:265–290.

[29] Lewis C, Long TAF: Courtship and reproduction in carybdea sivickisi (Cnidaria: cubozoa). Mar Biol 2005, 147:477–483.

[30] Nath RD, : The jellyfish cassiopea exhibits a sleep-like state. Curr Biol 2017, 27:2984–2990.e3. 28943083 10.1016/j.cub.2017.08.014 PMC5653286

[31] Sinigaglia C, : Pattern regulation in a regenerating jellyfish. Elife 2020, 9, e54868. 32894220 10.7554/eLife.54868 PMC7524552

[32] Abrams MJ, Basinger T, Yuan W, Guo C-L, Goentoro L: Self-repairing symmetry in jellyfish through mechanically driven reorganization. Proc Natl Acad Sci USA 2015, 112:E3365–E3373. 26080418 10.1073/pnas.1502497112 PMC4491739

[33] Condon RH, : Recurrent jellyfish blooms are a consequence of global oscillations. Proc Natl Acad Sci USA 2013, 110:1000–1005. 23277544 10.1073/pnas.1210920110 PMC3549082

[34] Graham WM, : Linking human well-being and jellyfish: ecosystem services, impacts, and societal responses. Front Ecol Environ 2014, 12:515–523.

[35] Hays GC, Doyle TK, Houghton JDR: Aparadigmshift in the trophic importance of jellyfish? Trends Ecol Evol 2018, 33:874–884. 30245075 10.1016/j.tree.2018.09.001

[36] Costello JH, : The hydrodynamics of jellyfish swimming. Ann Rev Mar Sci 2021, 13:375–396. 10.1146/annurev-marine-031120-091442 32600216

[37] Houliston E, Momose T, Manuel M: Clytia hemisphaerica: a jellyfish cousin joins the laboratory. Trends Genet 2010, 26:159–167. 20227783 10.1016/j.tig.2010.01.008

[R38] Chari T, : Whole-animal multiplexed single-cell RNA-seq reveals transcriptional shifts across Clytia medusa cell types. Sci Adv 2021, 7, eabh1683. 34826233 10.1126/sciadv.abh1683 PMC8626072

[39] Ahrens MB, Engert F: Large-scale imaging in small brains. Curr Opin Neurobiol 2015, 32:78–86. 25636154 10.1016/j.conb.2015.01.007 PMC4955592

[40] Masuda-Ozawa T, : siRNA-mediated gene knockdown via electroporation in hydrozoan jellyfish embryos. Sci Rep 2022, 12, 16049. 36180523 10.1038/s41598-022-20476-1 PMC9525680

[41] Momose T, : High doses of CRISPR/Cas9 ribonucleoprotein efficiently induce gene knockout with low mosaicism in the hydrozoan Clytia hemisphaerica through microhomology-mediated deletion. Sci Rep 2018, 8, 11734. 30082705 10.1038/s41598-018-30188-0 PMC6078951

[42] Lechable M, : An improved whole life cycle culture protocol for the hydrozoan genetic model Clytia hemisphaerica. Biol. Open 2020, 9. 10.1242/bio.051268 PMC7657476 32994186

[43] Carrier T, Reitzel A, Heyland A: Evolutionary ecology of marine invertebrate larvae. Oxford University Press; 2017.

[44] Khalturin K, : Medusozoan genomes inform the evolution of the jellyfish body plan. Nat. Ecol. Evol 2019, 3:811–822 30988488 10.1038/s41559-019-0853-y

[45] Romanes GJ: Jelly-fish, star-fish, and sea urchins: being a research on primitive nervous systems. New York: D. Appleton; 1885.

[46] Satterlie RA: Neuronal control of swimming in jellyfish: a comparative story. Can J Zool 2002, 80:1654–1669.

[47] Koizumi O, : The nerve ring in cnidarians: its presence and structure in hydrozoan medusae. Zoology 2015, 118:79–88 25498132 10.1016/j.zool.2014.10.001

[48] Satterlie RA, Spencer AN: Neuronal control of locomotion in hydrozoan medusae: a comparative study. J Comp Physiol 1983, 150:195–206.

[49] Satterlie RA: Do jellyfish have central nervous systems? J Exp Biol 2011, 214:1215–1223. 21430196 10.1242/jeb.043687

[50] Mackie GO: Central circuitry in the jellyfish Aglantha digitale IV. Pathways coordinating feeding behaviour. J Exp Biol 2003, 206:2487–2505. 12796463 10.1242/jeb.00450

[51] Mackie GO: Central neural circuitry in the jellyfish Aglantha: a model ‘simple nervous system’. Neurosignals 2004, 13:5–19. 15004422 10.1159/000076155

[52] Passano LM: Pacemakers and activity patterns in medusae: homage to romanes. Am Zool 1965, 5:465–481. 14345251 10.1093/icb/5.3.465

[53] Corrales-Ugalde M, Sutherland KR: Fluid mechanics of feeding determine the trophic niche of the hydromedusa *Clytia gregaria*. Limnol. Oceanogr. lno 2020, 11653, 10.1002/lno.11653.

[54] Mackie GO, Meech RW, Spencer AN: A new inhibitory pathway in the jellyfish Polyorchis penicillatus. Can J Zool 2012, 90:172–181.

[55] Satterlie RA: Control of swimming in the hydrozoan jellyfish Aequorea victoria: subumbrellar organization and local inhibition. J Exp Biol 2008, 211:3467–3477. 18931319 10.1242/jeb.018952

[56] King MG, Spencer AN: The involvement of nerves in the epithelial control of crumpling behavior in a hydrozoan jellyfish. J Exp Biol 1981, 94:203–218.

[57] Singla CL: Statocysts of hydromedusae. Cell Tissue Res 1975, 158:391–407. 238743 10.1007/BF00223835

[58] Arkett SA, Mackie G, Meech R: Hair cell mechanoreception in the jellyfish Aglantha digitale. J Exp Biol 1988.

[59] Singla CL: Fine structure of the sensory receptors of Aglantha digitale (Hydromedusae: trachylina). Cell Tissue Res 1983, 231:415–425. 6133627 10.1007/BF00222191

[60] Shimomura O: The discovery of aequorin and green fluorescent protein. J Microsc 2005, 217:3–15. 10.1111/j.0022-2720.2005.01441.x 15655058

[61] Satterlie RA: Central generation of swimming activity in the hydrozoan jellyfish Aequorea aequorea. J Neurobiol 1985, 16:41–55. 2859349 10.1002/neu.480160105

[62] Spencer AN, Satterlie RA: Electrical and dye coupling in an identified group of neurons in a coelenterate. J Neurobiol 1980, 11:13–19. 6101612 10.1002/neu.480110103

[63] Satterlie RA: Control of swimming in the hydrozoan jellyfish Aequorea aequorea: direct activation of the subumbrella. J Neurobiol 1985, 16:211–226. 2861247 10.1002/neu.480160306

[64] Romanes III GJ: Preliminary observations on the locomotor system of medusæ. Proc Roy Soc Lond 1875, 24:143–151.

[65] Arkett SA, Spencer AN: Neuronal mechanisms of a hydromedusan shadow reflex. J Comp Physiol 1986, 159:215–225.

[66] Anderson P, Mackie G: Electrically coupled, photosensitive neurons control swimming in a jellyfish. Science 1977, 197:186–188. 17918 10.1126/science.17918

[67] Arkett SA, Spencer AN: Neuronal mechanisms of a hydromedusan shadow reflex. J Comp Physiol 1986, 159:201–213.

[68] Quiroga Artigas G, : A gonad-expressed opsin mediates light-induced spawning in the jellyfish Clytia. Elife 2018, 7, e29555. 29303477 10.7554/eLife.29555 PMC5756024

[R69] Artigas GQ, : A G protein–coupled receptor mediates neuropeptide-induced oocyte maturation in the jellyfish Clytia. PLoS Biol 2020, 18, e3000614. 32126082 10.1371/journal.pbio.3000614 PMC7053711

[70] Takeda N, : Identification of jellyfish neuropeptides that act directly as oocyte maturation-inducing hormones. Development 2018, 145, dev156786. 29358214 10.1242/dev.156786

[71] Garm A, Ekström P: Chapter 2 - evidence for multiple photosystems in jellyfish. . International review of cell and molecular biology, vol. 280. Academic Press; 2010:41–78. 20797681 10.1016/S1937-6448(10)80002-4

[72] Spencer AN: The parameters and properties of A group of electrically coupled neurones in the central nervous system of A Hydrozoan jellyfish. J Exp Biol 1981, 93:33–50.

[73] Anderson DJ, Adolphs R: A framework for studying emotions across species. Cell 2014, 157:187–200. 24679535 10.1016/j.cell.2014.03.003 PMC4098837

[74] Donaldson S, Mackie GO, Roberts A: Preliminary observations on escape swimming and giant neurons in Aglantha digitale (Hydromedusae: trachylina). Can J Zool 1980, 58:549–552.

[75] Mackie GO, Meech RW: Central circuitry in the jellyfish Aglantha digitale: I. The relay system. J Exp Biol 1995, 198:2261–2270. 9320176 10.1242/jeb.198.11.2261

[76] Mackie GO, Meech RW: Central circuitry in the jellyfish Aglantha digitale: II. The ring giant and carrier systems. J Exp Biol 1995, 198:2271–2278. 9320190 10.1242/jeb.198.11.2271

[77] Meech RW, Mackie GO: Synaptic potentials and threshold currents underlying spike production in motor giant axons of Aglantha digitale. J Neurophysiol 1995, 74:1662–1670. 8989402 10.1152/jn.1995.74.4.1662

[78] Mackie GO, Meech RW: Separate sodium and calcium spikes in the same axon. Nature 1985, 313:791–793. 2858055 10.1038/313791a0

[79] Hyman LH: Observations and experiments on the physiology of medusae. Biol. Bull 1940, 79:282–296.

[80] Horridge GA: The nerves and muscles of medusae: II. Geryonia proboscidalis eschscholtz. J Exp Biol 1955, 32:555–568.

[81] Miglietta MP, : Approaches to the ethology of hydroids and medusae (Cnidaria, Hydrozoa). Sci Mar 2000, 64:63–71.

[82] Mackie GO, Singla CL: Neurobiology of stomotoca. I. Action systems. J Neurobiol 1975, 6:339–356. 241777 10.1002/neu.480060402

[83] Weissbourd B, : Functional modules within a distributed neural network control feeding in a model medusa. bioRxiv 2021, 432372, 10.1101/2021.02.22.432372. 2021.02.22.

[84] Larson RJ, Mills CE, Harbison GR: *In situ* foraging and feeding behaviour of narcomedusae (Cnidaria: Hydrozoa). J Mar Biol Assoc U K 1989, 69:785–794.

[85] Tachibana K, Matsumoto M, Minowa A, Deguchi R: Isolation and characterization of feeding-deficient strains in inbred lines of the Hydrozoan jellyfish cladonema pacificum. Zool Sci (Tokyo) 2020, 37:263. 10.2108/zs190122 32549540

[86] Marques AC, Collins AG: Cladistic analysis of Medusozoa and cnidarian evolution. Invertebr Biol 2005, 123:23–42.

[87] Leclère L, Röttinger E: Diversity of Cnidarian muscles: function, anatomy, development and regeneration. Front Cell Dev Biol 2017, 4:157. 28168188 10.3389/fcell.2016.00157 PMC5253434

[88] Singla CL: Fine structure of the neuromuscular system of Polyorchis penicillatus (Hydromedusae, Cnidaria). Cell Tissue Res 1978, 193:163–174. 31237 10.1007/BF00221609

[89] Denker E, Manuel M, Leclère L, Guyader HL, Rabet N: Ordered progression of nematogenesis from stem cells through differentiation stages in the tentacle bulb of Clytia hemisphaerica (Hydrozoa, Cnidaria). Dev Biol 2008, 315:99–113. 18234172 10.1016/j.ydbio.2007.12.023

